# LncRNA *MIR31HG* Drives Oncogenicity by Inhibiting the Limb-Bud and Heart Development Gene (*LBH*) during Oral Carcinoma

**DOI:** 10.3390/ijms22168383

**Published:** 2021-08-04

**Authors:** Kuo-Wei Chang, Wan-Wen Hung, Chung-Hsien Chou, Hsi-Feng Tu, Shi-Rou Chang, Ying-Chieh Liu, Chung-Ji Liu, Shu-Chun Lin

**Affiliations:** 1 Department of Dentistry, College of Dentistry, National Yang Ming Chiao Tung University, Taipei 112, Taiwan; ckcw@nycu.edu.tw (K.-W.C.); hsifeng@gmail.com (H.-F.T.); cjliu3229@gmail.com (C.-J.L.); 2 Institute of Oral Biology, College of Dentistry, National Yang Ming Chiao Tung University, Taipei 112, Taiwan; vivian25wen@gmail.com (W.-W.H.); michaelchou0806@gmail.com (C.-H.C.); s4103052122@gmail.com (S.-R.C.); yingchieh12@gmail.com (Y.-C.L.); 3 Department of Stomatology, Taipei Veterans General Hospital, Taipei 112, Taiwan; 4 Department of Dentistry, National Yang Ming Chiao Tung Hospital, Yilan 260, Taiwan; 5 Department of Dentistry, Taipei MacKay Memorial Hospital, Taipei 104, Taiwan

**Keywords:** carcinoma, *LBH*, *MIR31HG*, oral, precancer

## Abstract

The *miR-31* host gene (*MIR31HG*) encodes a long non-coding RNA (LncRNA) that harbors *miR-31* in its intron 2; *miR-31* promotes malignant neoplastic progression. Overexpression of *MIR31HG* and of *miR-31* occurs during oral squamous cell carcinoma (OSCC). However, the downstream effectors modulated by *MIR31HG* during OSCC pathogenesis remain unclear. The present study identifies up-regulation of *MIR31HG* expression during the potentially premalignant disorder stage of oral carcinogenesis. The potential of *MIR31HG* to enhance oncogenicity and to activate Wnt and FAK was identified when there was exogenous *MIR31HG* expression in OSCC cells. Furthermore, OSCC cell subclones with *MIR31HG* deleted were established using a Crispr/Cas9 strategy. RNA sequencing data obtained from cells expressing *MIR31HG*, cells with *MIR31HG* deleted and cells with *miR-31* deleted identified 17 candidate genes that seem to be modulated by *MIR31HG* in OSCC cells. A TCGA database algorithm pinpointed *MMP1*, *BMP2* and Limb-Bud and Heart development (*LBH*) as effector genes controlled by *MIR31HG* during OSCC. Exogenous *LBH* expression decreases tumor cell invasiveness, while knockdown of *LBH* reverses the oncogenic suppression present in *MIR31HG* deletion subclones. The study provides novel insights demonstrating the contribution of the *MIR31HG-LBH* cascade to oral carcinogenesis.

## 1. Introduction

Head and neck squamous cell carcinoma (HNSCC), which includes oral SCC (OSCC), is one of the major causes of cancer death worldwide [1,2,3]. The survival rate of HNSCC has not been remarkably improved over past decades due mainly to our limited mechanistic understanding of relapse, drug resistance and metastasis [4,5]. To prevent progression from oral potentially malignant disorder (OPMD) to a full neoplasm is also likely way to improve the survival rate of individuals at risk [6,7]. Long non-coding RNAs (LncRNAs) and miRNAs are non-coding RNAs that are crucial to the regulation of pathogenesis, including the neoplastic process of HNSCC [8]. Our series of studies have identified the oncogenic roles that *miR-31* plays in OSCC by targeting suppressor molecules; these molecules control the induction of hypoxia, the induction of stemness, the creation of metabolic aberrances, and an increase in susceptibility to DNA damage [1,9,10,11]. The up-regulation of *miR-31* occurs early during the OPMD stage, and this aberrance is a prognostic predictor of this disorder [12,13]. In addition, *miR-31* has been validated as an OSCC marker in biofluids, including plasma and saliva [14,15]. A further elucidation of the effectors or co-players associated with *miR-31* should help to bring about further therapeutic advances.

It is known that LncRNAs play a range of versatile roles in physiological modulation [8]. In the nucleus, LncRNAs seem to be involved in reshaping the configuration of chromatin, guiding transcription factors to allow promoter activation, and acting as an isolator during inhibition of gene transcription [16]. In the cytosol, LncRNAs may interact with various molecules that modulate gene translation, as well as being able to complex with miRNA, which allows them to act as a sponge resulting in functional abrogation. *MIR31HG*, which localized on chromosome 9p21 and was previously defined LOC554202 locus, is a ~150-Kb gene that consists of 4 exons; it hosts *miR-31* in its intron 2 [16,17,18,19,20]. *MIR31HG* is transcribed as a 2148-bp LncRNA, and this molecule has been shown to play diverse roles in various malignancies [19,21,22,23,24,25,26,27,28,29,30,31]. As a competing endogenous RNA, *MIR31HG* binds with multiple miRNAs and this activity acts as a sink that can either enrich or repress malignancy [22,23,26,32,33]. In non-small cell lung cancer (NSCLC), an up-regulated *MIR31HG* activates the EGFR/AKT cascade resulting in resistance to Gefitinib [34]. *MIR31HG* also activates the Wnt/β-catenin cascades, which increases the aggressiveness of tumors [35]. Nuclear *MIR31HG* interacts with p16 to recruit polycomb proteins and this brings about p16 repression [18]. On the other hand, cytosolic *MIR31HG* interacts with YBX1 and this modulates the secretory phenotype [17]. In OSCC, *miR-31* induces hypoxia by inhibiting FIH, which then activates HIF1α [1]. Interestingly, *MIR31HG* acts together with *miR-31* to complex with HIF1α this then enriches the binding of HIF1α/p300 to hypoxia response elements [19]. *MIR31HG* also targets p21 in HNSCC, which promotes the cell cycle and inhibits apoptosis [36]. However, by way of contrast, *MIR31HG* has been found to acts as a proapoptotic factor by down-regulating malignancies via the hypermethylation of various promotors [37]. The functional roles and specific downstream effectors of *MIR31HG* during OSCC remain to be clarified.

Limb-Bud and Heart development (*LBH*) is a highly conserved transcriptional component originally found to regulate tissue development during early embryogenesis [38]. The Wnt-LBH regulatory axis is crucial for the maintenance of the basal lineage of mammary stem cells and the pathogenesis of the aggressive basal subtype of breast cancer [39,40]. In addition to the above, LBH also modulates Wnt-associated proliferation, apoptosis and tumorigenicity during breast carcinogenesis [41]. Furthermore, *LBH* functions as suppressor during nasopharyngeal carcinoma, prostate carcinoma and NSCLC [42,43,44,45]. The roles played by *LBH* in OSCC pathogenesis remain hitherto obscure. MMP1 and BMP2 are known to modulate cell activity and the tumor microenvironment, as well as being factors associated with OSCC relapse [5,46,47]. This study identifies the up-regulation of *MIR31HG* in cytobrushed OPMD samples. Using knockout and expression strategies, followed by bioinformatic analysis and functional validation, this study shows that *MIR31HG* modulates MMP1, BMP2, and LBH expression to augment OSCC oncogenicity.

## 2. Results

### 2.1. Up-Regulation of MIR31HG in OPMD and OSCC

**Figure 1:**
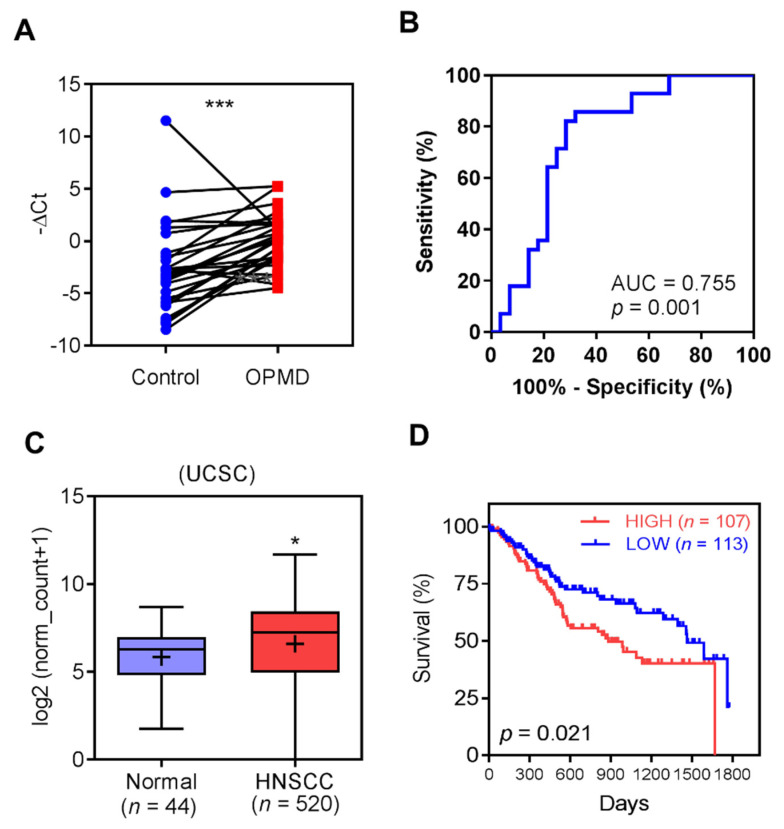
*MIR31HG* expression in brushed OPMD samples and the HNSCC dataset. (**A**) Before-after-blot demonstrating the increase in the −ΔCt values for *MIR31HG* obtained from samples of normal mucosa compared to those obtained from OPMD. (**B**) ROC curve of the −ΔCt of *MIR31HG* showing an accuracy of 0.76 when separating OPMD samples from control samples. (**C**) Increased *MIR31HG* expression in HNSCC tumors relative to normal tissues extracted from the TCGA dataset. (**D**) Kaplan–Meier survival analysis. This indicates that HNSCCs showing increased *MIR31HG* expression in the highest quadrant exhibits a worse prognosis than those with lower expression in the lowest quadrant. *, *p* < 0.05; ***, *p* < 0.001.

An analysis of the brushed samples showed an increase in *MIR31HG* expression for OPMD relative to that of the controls (Figure 1A; Table S1). ROC analysis revealed an accuracy of 0.76 when *MIR31HG* expression was used to separate OPMD samples from the control samples (Figure 1B). *MIR31HG* expression was not associated with the dysplasia state among the OPMD samples. The importance of *MIR31HG* to HNSCC was further specified using The Cancer Genome Atlas (TCGA) dataset, which confirmed *MIR31HG* up-regulation in tumors (Figure 1C). In addition, tumors that had *MIR31HG* expression levels within the highest quadrant had a worse survival than those that had *MIR31HG* expression levels within the lowest quadrant (Figure 1D).

### 2.2. Overexpression of MIR31HG Enhances Various Oncogenic Phenotypes

**Figure 2:**
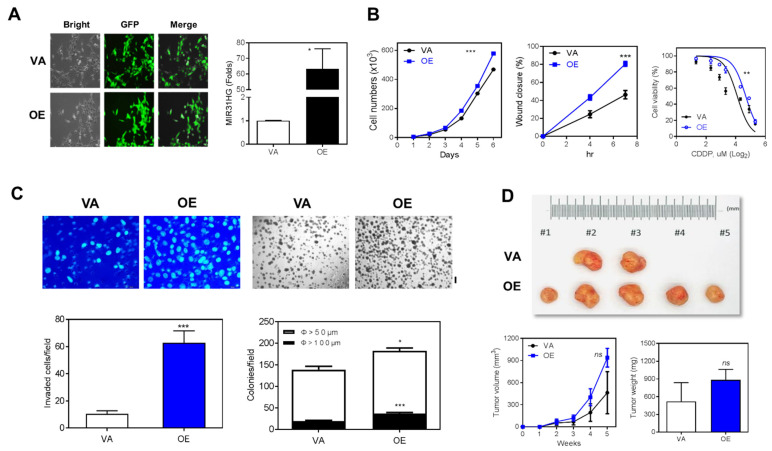
Exogenous *MIR31HG* overexpression increases the oncogenicity of SAS cells. (**A**) Left, stable cells with exogenous *MIR31HG* overexpression and control cells both exhibit green fluorescence indicating the presence of plasmid. Right, qRT-PCR analysis shows that there is increased *MIR31HG* expression in OE cells. (**B**) OE cells exhibit an increased proliferation (Left), increased wound closure rate (Middle) and higher cisplatin resistance (Right) relative to VA cells. (**C**) OE cells exhibit increased invasion (Upper Left) and higher colony formation ability (Upper Right) relative to VA cells. The lower panels show the calculation. Bar, 200 µM. (**D**) Subcutaneous tumorigenesis assay. Left, resected tumors from the sacrificed animals. Middle, the growth rate of the tumors over a 5-week period. Right, the weight of the resected tumors. VA, vector alone. OE, stable *MIR31HG* overexpression. *, *p* < 0.05; **, *p* < 0.01; ***, *p* < 0.001.

SAS cell line was successfully infected with lentivirus, and this resulted in overexpression of *MIR31HG* and GFP and this stable cell was designated OE, relative to the vector alone (VA) control (Table S2; Figure S1). A robust increase in *MIR31HG* expression was detected in the OE (Figure 2A). Proliferation rate, wound closure rate and cisplatin resistance increased in the OE (Figure 2B). Nevertheless, no change in sensitivity was found between OE and VA when other drugs were tested (Figure S2). Invasion and colony formation of the OE increased to different extents (Figure 2C). Thus, OE cell seemed to exhibit an increased trend towards tumorigenesis compared to VA cell (Figure 2D).

### 2.3. Overexpression of MIR31HG Activates the Wnt Pathway

**Figure 3:**
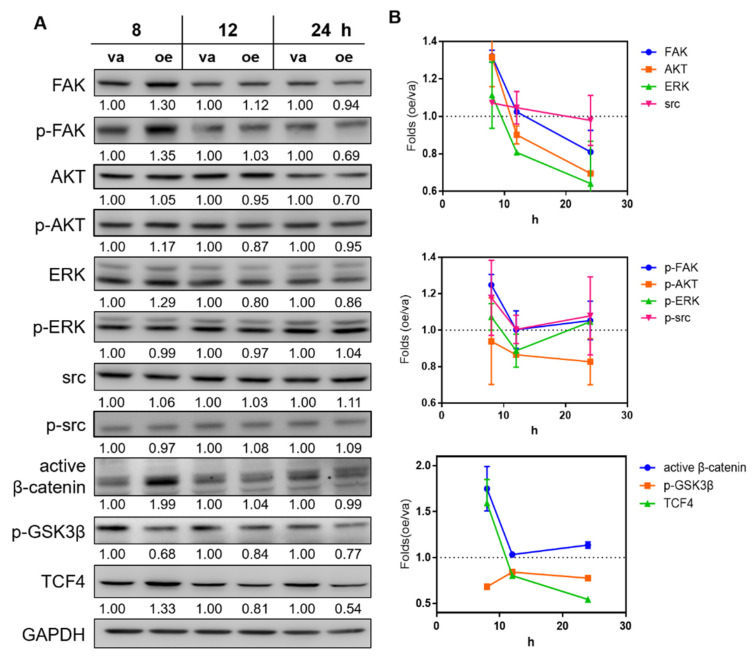
Signal activation after transient MIR31HG expression in SAS cells. (**A**) A representative Western blot analysis showing the expression of various signal factors after transient transfection with the MIR31HG plasmid for 8, 12, and 24 h. The numbers are normalized values. (**B**) The changes of signal protein levels over the time course following transfection. Upper, FAK, AKT, ERK, and src; Middle, p-FAK, p-AKT, p-ERK, and p-src; Lower, active β-catenin, p-GSK3β and TCF4. va, vector alone; oe, transient MIR31HG overexpression.

To pinpoint the influence of *MIR31HG* on various signals, a smaller plasmid was constructed that was able to transiently transfect cells (Figure S3; Table S2). SAS cells were transiently transfected to overexpress *MIR31HG*. The *MIR31HG* cells and control vector cells were designated OE and VA, respectively. Western blot analysis at 8 h after transfection showed increased expression of FAK/p-FAK, active β-catenin and TCF4, as well as decreased expression of p-GSK3β expression (Figure 3A,B). Over the time course studied activation of AKT, ERK or src was not prominent (Figure 3B).

### 2.4. Deletion of MIR31HG Reduces Oncogenic Phenotypes

**Figure 4:**
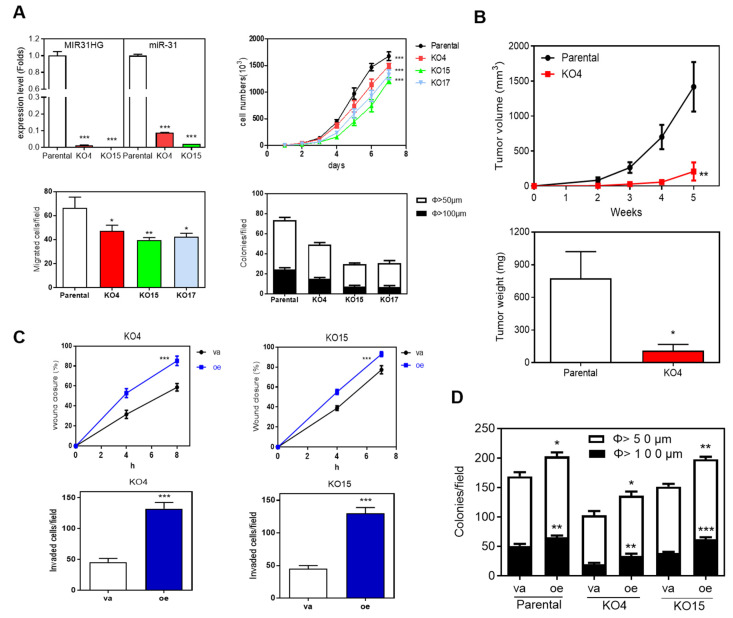
*MIR31HG* overexpression rescues the suppression brought about by the knockout of *MIR31HG* in SAS cells. (**A**) Association between *MIR31HG* knockout and decreased oncogenicity. Upper Left, the decreased expression of *MIR31HG* and *miR-31* in the knockout cell subclones KO4 and KO15. Upper Right, Lower Left, and Lower Right, the decreased growth, invasion, and colony formation in the knockout cell subclones KO4, KO15, and KO17 relative to parental cells, respectively. (**B**) Subcutaneous tumorigenesis assay. Upper, the growth curve of tumors over a 5-week period. Lower, the weight of the resected tumors. (**C**) Using the KO4 (left panel) and KO15 (right panel) cell subclones, transient *MIR31HG* overexpression increases the wound closure rate (upper panels) and tumor cell invasion (lower panels). (**D**) Anchorage-independent colony formation assay. In the parental cells, the KO4 cell subclone and the KO15 cell subclone, transient *MIR31HG* overexpression increases colony formation. va, vector alone, oe, transient *MIR31HG* overexpression. *, *p* < 0.05; **, *p* < 0.01; ***, *p* < 0.001.

SAS cell subclones were established after transfecting cells with the appropriate plasmids followed by selection. PCR analysis revealed that there was homozygous deletion of *MIR31HG* in the KO4, KO15 and KO17 subclones (Figure S4). Sequencing confirmed that the deletions in the subclones spanned exon 1 to exon 4 of *MIR31HG.* qRT-PCR analysis confirmed a drastic decrease in both *MIR31HG* and *miR-31* expression in the three subclones (Figure 4A, upper left). The deletions were associated with decreased proliferation, decreased migration and reduced colony formation (Figure 4A). The tumorigenic effect of the KO4 subclone were markedly decreased compared with that of the parental SAS cells (Figure 4B). When subclones KO4 and KO15 underwent exogenous overexpression of *MIR31HG*, this increased both the wound closure rate and invasion (Figure 4C). In addition, it was found the decreased colony formation capability of KO4 and KO15 was rescued by transient *MIR31HG* overexpression (Figure 4D).

### 2.5. Identification of Downstream Effectors of MIR31HG

**Figure 5:**
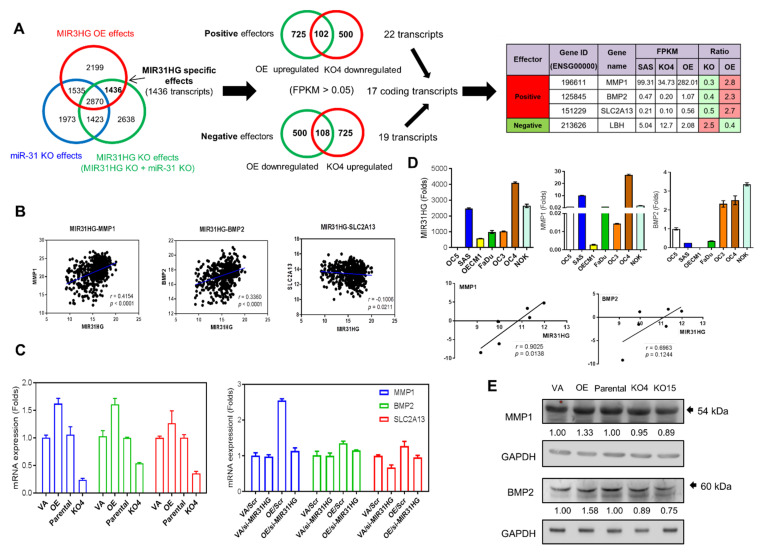
Identification of potential *MIR31HG* effectors in SAS cells. (**A**) The strategy using RNASeq data. Left, the identification of *MIR31HG* specific effectors. Middle, the identification of 22 positive and 19 negative effectors potentially modulated by *MIR31HG*. Specifically, 17 coding transcripts were potentially modulated by *MIR31HG*. Right, the expression profile of *MMP1*, *BMP2*, *SLC2A13*, and LBH, the genes selected for further analysis. (**B**) The correlation between *MIR31HG* expression and the expression of *MMP1*, *BMP2*, and *SCL2A13* in HNSCC tumors of the TCGA dataset. (**C**) qRT-PCR analysis reveals that the level of *MMP1*, *BMP2* and *SCL2A13* mRNA expression in SAS cells with either *MIR31HG* overexpression or *MIR31HG* deletion (left), as well as in cells that had been treated with si-*MIR31HG* or scramble (right). (**D**) Upper, qRT-PCR analysis to show *MIR31HG*, *MMP1*, and *BMP2* mRNA expression levels in various OSCC cell lines. Lower, a correlation is found between the expression of *MIR31HG* and *MMP1*, but not between *MIR31HG* and *BMP2*. (**E**) Western blot analysis. This shows an increase in MMP1 and BMP2 expression in stable cells with exogenous *MIR31HG* overexpression, and slightly decreased MMP1 and BMP2 expression in *MIR31HG* knockout cell subclones. VA or va, vector alone. OE, stable *MIR31HG* overexpression. oe, transient *MIR31HG* overexpression. Scr, scramble.

To pinpoint the downstream effectors specific for *MIR31HG*, this study also established a *miR-31* knockout cell subclone (designated *miR-31*KO) from SAS cells. RNASeq was carried out on OE, KO4, and *miR-31*KO cells. A total of 1436 transcripts were present in both the OE and KO sequencing datasets, but not present in the *miR-31*KO dataset; these were considered potential *MIR31HG* specific effectors (Figure 5A, Left). Among these, 102 positive effectors and 108 negative effectors were identified. Using an FPKM of more than 0.05 as the screening threshold, 22 positive transcripts and 19 negative transcripts were ultimately identified to be good candidates for *MIR31HG* specific effectors (Figure 5A, Middle; Table S3). After excluding pseudogenes, non-coding RNAs and various uncertain transcripts, 17 protein coding transcripts were finally retrieved as candidate effectors of *MIR31HG* in SAS cells (Figure 5A, Middle; Table S4). *MMP1*, *BMP2*, *SLC2A13*, and LBH, all of which exhibited conspicuous changes in expression level, were further tested (Figure 5A, Right). A positive correlation between *MIR31HG* expression and the expression of *MMP1* and *BMP2*; and a negative correlation between *MIR31HG* expression and SLC2A13 expression were identified in HNSCC samples using the TCGA dataset (Figure 5B; Figure S5). *MMP1*, *BMP2* and *SCL2A13* were up-regulated in OE cells and down-regulated in the KO4 cell subclone (Figure 5C, Left). In VA, *MMP1*, *BMP2*, and *SCL2A13* expression was slightly reduced by the knockdown of *MIR31HG*. However, in OE cells, up-regulated *MMP1*, *BMP2* and *SCL2A13* expression was more conspicuously decreased by the knockdown of *MIR31HG* (Figure 5C, Right). In the GSE37991 OSCC database, *MMP1* and *BMP2* expression was also up-regulated in tumors. The *SCL2A13* study was terminated due to the discrepancies in tissues and cells. In OSCC cell lines, a positive correlation between *MIR31HG* expression and the expression of *MMP1* was noted (Figure 5D). Western blot analysis revealed an increased MMP1 and BMP2 protein expression in OE cells, and a slight decrease in MMP1 and BMP2 protein expression in KO4 and KO15 cells (Figure 5E). The phenotypic impacts of *MMP1* were further analyzed using knockdown of expression. The knockdown of *MMP1* did not alter the expression of *MIR31HG*, or did it affect the proliferation/migration of SAS cells (Figure S6A–C). Nonetheless it did reduce invasion and colony formation by SAS cells (Figure S6D,E). HNSCC in the TCGA dataset that had the highest levels of *MMP1* expression also exhibited a trend to having a worse prognosis compared to the contrasting quadrant (Figure S6F).

### 2.6. LBH Is a Negative Effector of MIR31HG

**Figure 6:**
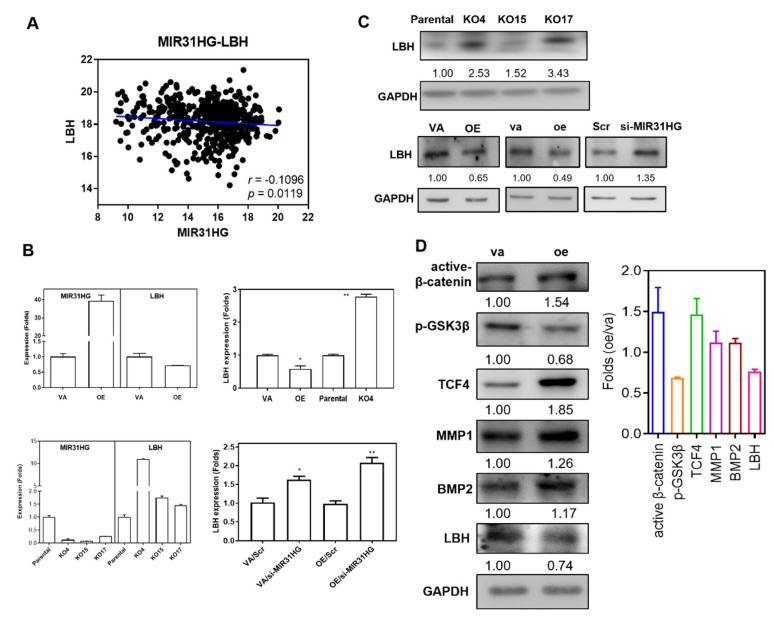
*LBH* is a negative effector of *MIR31HG*. (**A**) A weakly negative correlation between expression of *MIR31HG* and *LBH* can be seen in HNSCC tumors from the TCGA dataset. (**B**) qRT-PCR analysis. Upper, decreased *LBH* expression in *MIR31HG* overexpression cells, and increased *LBH* expression in the *MIR31HG* knockout cell subclone. Lower Left, decreased *MIR31HG* expression and increased *LBH* mRNA expression in the *MIR31HG* knockout cell subclones. Lower Right, knockdown of *MIR31HG* increases *LBH* expression in both VA and OE cells. At least duplicate analysis. (**C**) Western blot analysis. Upper, increased LBH expression in *MIR31HG* knockout cell subclones. Lower, both stable and transient *MIR31HG* overexpression decreases LBH protein expression, whereas knockdown of *MIR31HG* increases LBH protein expression. (**D**) Western blot analysis. Left, a representative analysis. Transient *MIR31HG* overexpression activates Wnt signal molecules, increases MMP1 and BMP2 expression, as well as decreasing LBH expression. Right, quantification of duplicate or triplicate analysis. VA or va, vector alone. OE, stable *MIR31HG* overexpression. oe, transient *MIR31HG* overexpression. Scr, scramble. *, *p* < 0.05; **, *p* < 0.01.

In the TCGA HNSCC dataset, a reverse correlation between *MIR31HG* expression and *LBH* expression was found (Figure 6A). In OE cells, *MIR31HG* expression increased, while the *LBH* mRNA expression decreased. By way of contrast, in the KO4 cell subclone, *LBH* mRNA expression increased (Figure 6B, Upper). Furthermore, *LBH* mRNA expression was also up-regulated in the KO15 and KO17 subclones (Figure 6B, Lower Left). In both the parental SAS cells and OE cells, the knockdown of *MIR31HG* increased *LBH* mRNA expression (Figure 6B, Lower Right). Similarly, in the *MIR31HG* KO cell subclones, *LBH* protein levels increased to different extents (Figure 6C, Upper). Transient or stable *MIR31HG* overexpression decreased *LBH* protein expression, whereas knockdown of *MIR31HG* expression increased *LBH* protein expression (Figure 6C, Lower). Western blot analysis further confirmed at 8 h after transient *MIR31HG* transfection that there was activation of Wnt pathway molecules and up-regulation of MMP1 and BMP2; these changes were accompanied by LBH down-regulation (Figure 6D). A positive correlation between *LBH* expression and the expression of several Wnt-associated molecules, especially TCF4 and TCF7, could be noted in TCGA HNSCC dataset (Figure S7).

### 2.7. The MIR31HG Associated Phenotypes Are Attenuated by LBH

**Figure 7:**
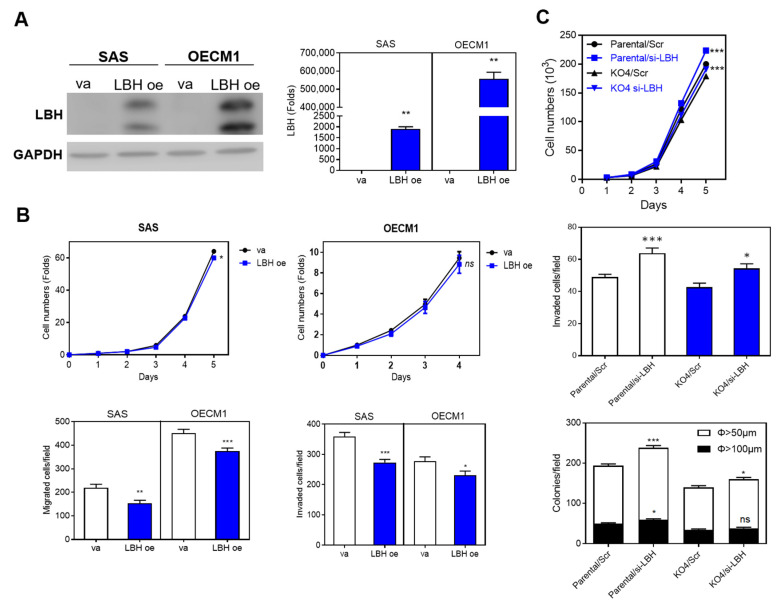
*LBH* suppresses the migration and invasion of OSCC cells. (**A**) Transient LBH overexpression in OSCC cells. Left, Western blot analysis. Only very brief exposure was carried out to acquire the images without a burn-through effect, this means that endogenous LBH expression is barely detectable. Right, qRT-PCR analysis shows increased *LBH* mRNA expression following transient overexpression. (**B**) Upper, proliferation; Lower Left, migration; Lower Right, invasion. The *LBH* expression in OSCC cells decreases migration and invasion by OSCC cells, but it does not affect proliferation of OSCC cells. (**C**) Knockdown of *LBH* reverts the phenotypes that resulted from *MIR31HG* deletion. Upper, proliferation; Middle, migration; Lower, anchorage-independent colony formation. va, vector alone; *LBH* oe, transient *LBH* overexpression. *, *p* < 0.05; **, *p* < 0.01; ***, *p* < 0.001.

Transient transfection of the *LBH* plasmid resulted in a tremendous overexpression of *LBH* protein in OSCC cells (Figure 7A; Figure S8; Table S2). This caused almost no effect on the proliferation of the OSCC cells (Figure 7B, Upper), but it did reduce the migration and invasion of the OSCC cells (Figure 7B, Lower). In addition, the decreased proliferation, invasion and colony formation capability of the KO4 cell subclone was reversed by knockdown of *LBH* (Figure 7C).

## 3. Discussion

Both *MIR31HG* and *miR-31* has been found to be up-regulated in HNSCC and in OSCC [14,19]. In addition, the up-regulation of *miR-31* is known to begin during the OPMD stage [12,13] that precedes the full establishment of OSCC. Sampling of OPMD is somewhat more difficult than OSCC as eradication by means of other than surgical resection remains a therapeutic option [7,48]. As a result, this study analyzes brushed samples from OPMD patients. The up-regulation of genes in OPMD samples relative to control samples when identified by this minimally invasive approach suggests that *MIR31HG* up-regulation occurs early in oral carcinogenesis. Extension of *MIR31HG* analysis to saliva testing should facilitate the development of non-invasive diagnostic approach for at risk patients [15,49]. To decipher the prognostic implication of *MIR31HG* up-regulation in OPMD will help to determine the usefulness of *MIR31HG* expression testing when deciding an appropriate interception technique [12].

Studies have shown that both activation of a SP1 response element and the methylation status of the LOC554202 promoter independently may affect *MIR31HG* expression and *miR-31* expression [23,37]. Our previous study has shown that the EGFR/AKT/CEBPβ cascade activates the LOC554202 promoter bringing about *miR-31* up-regulation in OSCC cells [50]. It is likely that this signal axis also underlies the *MIR31HG* up-regulation in OPMD and OSCC. The present study confirms the potent oncogenic induction is present in both stable and transient *MIR31HG* overexpression systems. To acquire the appropriate reverse insights, we have used a Crispr/Cas9 editing approach to knockout *MIR31HG*. As there might be various *MIR31HG* isoforms owing to alternative splicing [51], we designed a double cleavage system to delete the gene completely from the genome. Multiple cell subclones that had undergone deletion of an ~150-Kb genome sequence and these exhibited a consistent reduction in oncogenicity. The deletion of such a long sequence segment by means of Crispr/Cas 9 system was surprising. However, using a similar strategy, deletion of other long spans, up to one hundred Kbs of genomic sequence, have been carried out in other studies [52]. It should be noted that 9p21 is a hotspot locus for gene deletion in HNSCC and many other types of malignancies [20], and it seems likely that the chromosomal structure or molecular apparatus close to *MIR31HG* may facilitates these gene editing events. As 9p21 is also a gene desert, it has been suggested to be a nodal region for gene interaction [53]; therefore, additional geographic or functional impacts secondary to the structural disruption in this region needs investigation. Since unequivocal oncogenic suppression appears in multiple *MIR31HG* knockout cell subclones, and this suppression was rescued by *MIR31HG* overexpression, the cause-effect relationship between *MIR31HG* and oral carcinogenesis is very strongly supported by our knockout data.

This study has identified a *MIR31HG* induced differential gene expression profile that is independent of *miR-31*. MMP1 and BMP2, which are prognostic factors of HNSCC [5,46,47], and are up-regulated by *MIR31HG* at the transcription level. During the senescence induced by oncogenic stimulation, cytosolic *MIR31HG* would seem to increase IL1A protein levels, which then transactivates a panel of secretory molecules such as *MMP1* [17]. Since concordance between *MIR31HG* expression and expression of these two secretory molecules has been noted in a HNSCC cohort, and it is known that these secreted molecules seem to be able to modify tumor plasticity via remote effects, this validation of their coordination provides further useful mechanistic insights. Strategies to prevent or abrogate against *MIR31HG* associated OPMD pathogenesis by targeting of *MMP1* or *BMP2* need to be considered [54]. The regulatory effects of *MIR31HG* on other annotated genes linked to oral pathogenesis also requires further investigation.

Many oncogenic signals seem to be activated by *MIR31HG* in tumor cells [34,35]. Our findings identify FAK and Wnt signaling elements as possible key factors related to *MIR31HG* expression [35]. This study also shows for the first time the inhibitory activity of *LBH* on invasion and colony formation by OSCC cells. The reversed correlation in expression between *MIR31HG* and *LBH* in the various cells and tumor cohorts, along with the reversed phenotypes found between *MIR31HG* and *LBH,* suggest a cause-effect situation. Although Wnt activation is responsible for *LBH* up-regulation [39,40,41], and the TCGA tissue datasets substantiate their positive association, the relevance of the relationship between *MIR31HG*-Wnt activation and the *LBH* down-regulation in OSCC remains to be addressed. A direct interaction between *MIR31HG* and *LBH* in nucleus, or trans-inactivation of the *LBH* promoter mediated by TCFs, are two possibilities and require further study. Our findings suggest that activation of FAK contributes to *LBH* expression, and this issue deserves investigation. As *MIR31HG* also affects the HIF1α and represses p21 in HNSCC [19,36], the overall effect seems to be one that brings advantages to OPMD or OSCC progression.

## 4. Materials and Methods

### 4.1. Subjects

Cytobrushed samples from 28 OPMD patients and their matched mucosa were collected at Taipei MacKay Memorial Hospital and National Yang Ming Chiao Tung University Hospital (Table S1). This study was approved by the appropriate ethics reviewing committees with approval numbers 18MMHIS187e and 2019A013, respectively. Libo specimen collection swabs (Iron Will, New Taipei City, Taiwan) were used to collect samples according to a previously used protocol [55]. Written informed consent was obtained from each patient prior to sampling.

### 4.2. Cell Lines

The OSCC cell lines OC3, OC4, OC5, SAS, OECM1, and FaDu, as well as hTERT immortalized normal oral keratinocytes, designated NOK, were cultured as previously described [2]. Small interference RNA oligonucleotides (Table S5) and their scramble (Scr) control were purchased from Ambion (Austin, TX, USA) or BioTools (New Taipei City, Taiwan). Unless specified, all other reagents were obtained from Sigma-Aldrich (St Louise, MO, USA).

### 4.3. qRT-PCR Analysis

TRI-reagent (Molecular Research Center, Cincinnati, OH) was used to isolate RNA from cells. TaqMan miRNA assay kits (Apply Biosystems, Waltham, MA, USA) were used to quantify the expression of *MIR31HG*, *miR-31, MMP1, BMP2, SLC2A13*, and LBH, while *GAPDH* or *RNU6B* were used as internal controls (Table S6). −ΔCt is the difference in threshold cycle number between the test gene and the internal control. −ΔΔCt is the difference in −ΔCt between the test group and the control group. 2^−ΔΔCt^ designates the differences in expression across the various groups [1].

### 4.4. Western Blotting

Cell lysates were subjected to Western blot analysis using various primary antibodies (Table S7) and their appropriate secondary antibodies (Table S8). The signals for the tested proteins were normalized against the signal for GAPDH to measure and compare expression [1].

### 4.5. Plasmid Construction and Overexpression

A ~2148-bp modified PCR product of the *MIR31HG* transcript with sticky ends was cloned into the pLV-EF1a-GFP lentiviral vector (Figure S1; Table S2) and the pcDNA 3.1(−) vector (Figure S3; Table S2) to allow stable cell lines to be established by lentiviral infection or transient overexpression by plasmid transfection, respectively [1]. In addition, a 342-bp PCR product amplified from the cDNA of the *LBH* gene was also cloned into pcDNA 3.1(−) plasmid to allow transient overexpression (Figure S8; Table S2). Transfectin (BioRad, Hercules, CA, USA) was used for all transfections.

### 4.6. The Implement of Clustered Regularly Interspaced Short Palindromic Repeat (Crispr)/Cas9 Approach for MIR31HG Deletion

The 5′sgRNA targeting exon 1 and the 3′ sgRNA targeting exon 4, cloned into the pRGEN_U6_sgRNA vector, was obtained from BioTools. The vectors containing the cloned DNA were then co-transfected with the pRGEN-Cas9-CMV vector into cells to express the two sgRNAs and Cas9, which resulted in the deletion of *MIR31HG* from the cell genome (Figure S4). After puromycin selection, single cells were selected by limited dilution, and were expanded to become cell subclones. DNA isolated from the cell subclones was subjected to PCR analysis to confirm the deletion of the target locus and the allelic status of the subclone (Figure S4). These PCR products were cloned into a bacteria vector, and plasmid DNAs from randomly selected bacterial colonies were sequenced to confirm that deletion at the *MIR31HG* locus had occurred [2].

### 4.7. Phenotypic and Tumorigenic Assays

Cell growth, wound closure, anchorage-independent colony formation, drug sensitivity, and transwell migration and transwell invasion assays were carried out according to previously published protocols [1,2]. For the migration and invasion assays, cell growth was arrested by treatment with 1 μM hydroxyurea. For the induction of subcutaneous xenografts, 5 × 10^5^ cells were injected into the flanks of nude mice. The resulting tumors had their longest (L) and shortest (S) diameters measured, and their volumes were assessed using the following formula: volume = 0.5LS^2^ [1,2]. This animal study was approved by the Institutional Animal Care and Use Committee of National Yang Ming University.

### 4.8. RNASeq

After removal of rRNA from total RNA, the remained RNA was fragmented, modified, and subjected to cDNA library construction. Specifically, 9 G/150 bp paired end sequencing was performed on an Illumina NovaSeq platform at Wegene Biotech (Taipei, Taiwan). The reads were aligned and the expression was quantified using cufflinks [56].

### 4.9. Statistics

Data are shown as mean ± SE. Mann–Whitney tests, *t-*tests, two-way ANOVA tests, linear correlation analysis and Kaplan–Meier survival analysis were performed. The tested genes in the HNSCC subset of TCGA database were analyzed using UCSC Xena Functional Genomics Explorer (https://xenabrowser.net/, accessed on 18 June 2021). *ns*, not significant and 1–3 asterisks (*, ** and ***) represent *p* < 0.05, *p* < 0.01 and *p* < 0.001, respectively.

## 5. Conclusions

Collectively, this study has identified the up-regulation of *MIR31HG* at an early stage of oral carcinogenesis. Oncogenic proteins MMP1 and BMP2, together with LBH, which suppresses tumor progression, have been found to act as downstream effectors of *MIR31HG*. To target *MIR31HG* is likely to have significant therapeutic efficacy due to the resulting concomitant modulation of multiple downstream effector genes.

## Data Availability

Not applicable.

## Supplementary Materials

The following are available online at https://www.mdpi.com/article/10.3390/ijms22168383/s1.

## Abbreviations

CRISPR	Clustered: regularly interspaced, short palindromic repeats
HNSCC	Head and neck squamous cell carcinoma
LBH	Limb-Bud and Heart development
LncRNA	Long non-coding RNA
NSCLC	Non-small cell lung cancer
OPMD	Oral potentially malignant disorder
OSCC	Oral squamous cell carcinoma
TCGA	The Cancer Genome Atlas
