# Supplementary material for: LncRNA MIR31HG Drives Oncogenicity by Inhibiting the Limb-Bud and Heart Development Gene (LBH) during Oral Carcinoma
Source: Int J Mol Sci. 2021 Aug 4;22(16):8383. doi: 10.3390/ijms22168383 (PMC8395036; doi:10.3390/ijms22168383)
Supplement: Supplementary file 1 [file ijms-22-08383-s001.zip › ijms-1323097-supplementary-new.pdf]

Supplementary materials

Supplementary figures

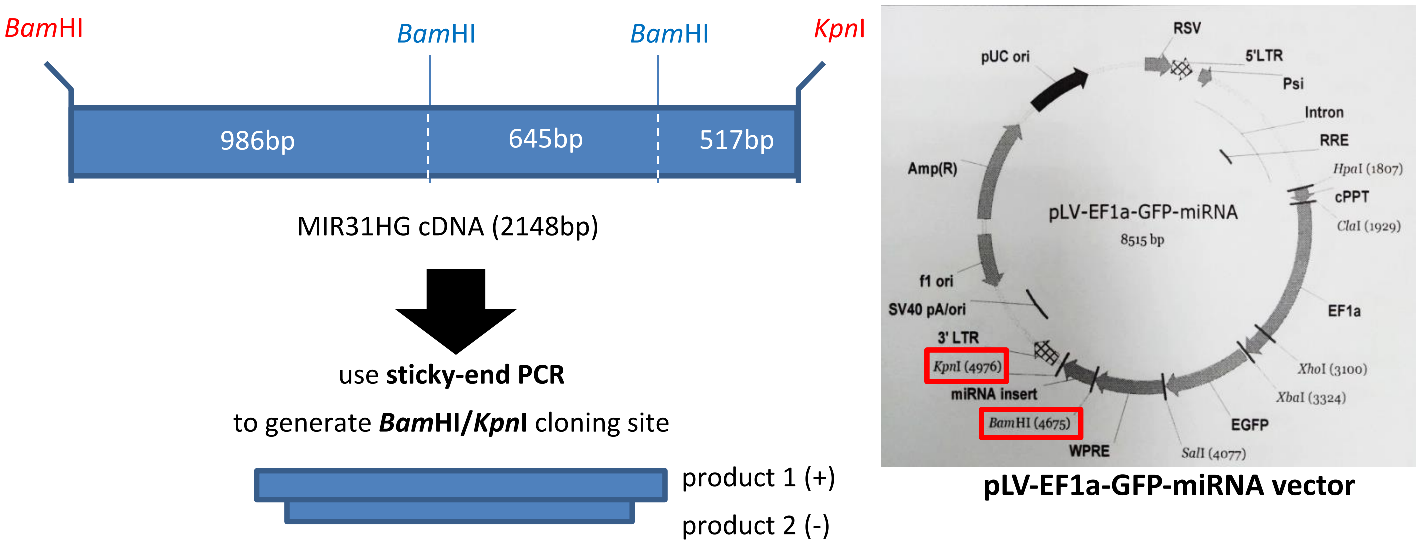

F1+R1 PCR product 1

GATCCAGGTTCCACGTCCGGCGCCTGGAG......ACAATAAATCCTTTGTGACCTT
TGTCGGTAC
CTAGGTCCAAGGTGCAGGCCGCGGACCTC......TGTTATTTAGGAAACACTGGAA
ACAGCCATG

F2+R2 PCR product 2

CAGGTTCCACGTCCGGCGCCTGGAG......ACAATAAATCCTTTGTGACCTTT
GTCG
GTCCAAGGTGCAGGCCGCGGACCTC......TGTTATTTAGGAAACACTGGAA
ACAGC

mix and re-anneal

one of re-annealing products with BamHI/KpnI sticky-end

GATCCAGGTTCCACGTCCGGCGCCTGGAG......ACAATAAATCCTTTGTGACCTT
TGTCGGTAC
GTCCAAGGTGCAGGCCGCGGACCTC......TGTTATTTAGGAAACACTGGAA
ACAGC

one of re-annealing products

CAGGTTCCACGTCCGGCGCCTGGAG......ACAATAAATCCTTTGTGACCTT
TGTCG
CTAGGTCCAAGGTGCAGGCCGCGGACCTC......TGTTATTTAGGAAACACTGGAA
ACAGCCATG

Figure S1. *MIR31HG* lentiviral plasmid. The strategy for the construction of the

MIR31HG lentiviral plasmid that was used to establish stable overexpression in cells.

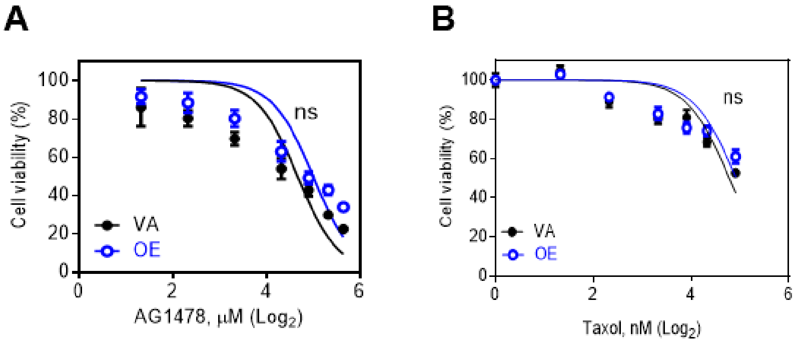

Figure S2. The dose-responses of OE and VA cells. (A) AG1478; (B) taxol.

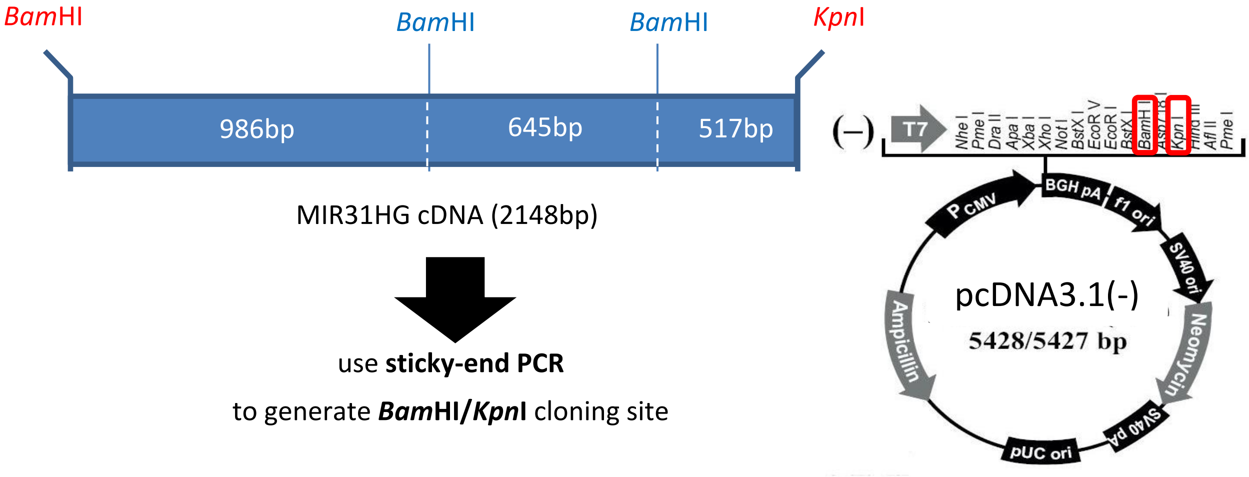

Figure S3. *MIR31HG* transient overexpression plasmid. The strategy for the construction of the *MIR31HG* plasmid for transient overexpression.

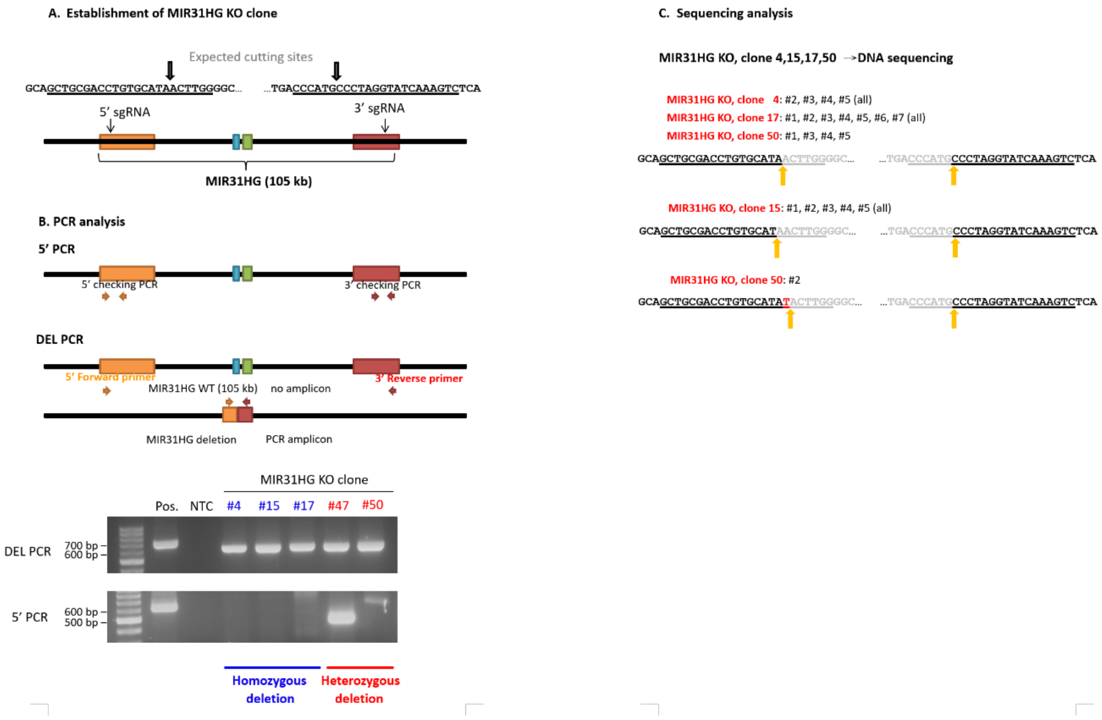

Figure S4. Construction of the *MIR31HG* Crispr/Cas9 plasmids and the establishment of stable cell subclones containing *MIR31HG* deletion. (A) The design of *MIR31HG* knockout. (B) PCR analysis. (C) Sequencing of the knockout cell subclones.

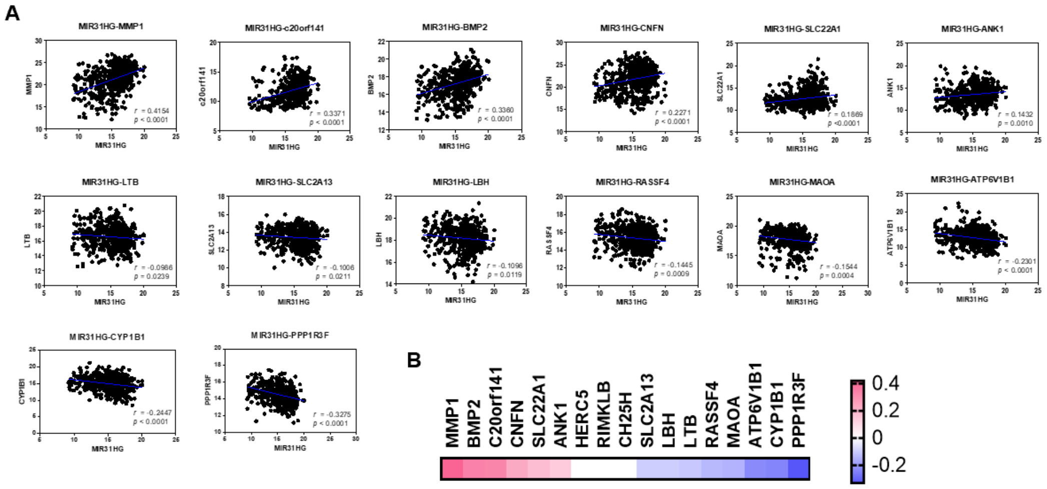

Figure S5. The association between *MIR31HG* expression and the expression of seventeen candidate genes in HNSCC tumors based on the TCGA dataset. (A) Individual analysis. (B) Heatmap to illustrate the correlations. The expression levels of a number of genes, but not of *CH25H*, *RIMKLB* and *HERC5*, are correlated with *MIR31HG* expression. Gradient bar, correlation coefficient.

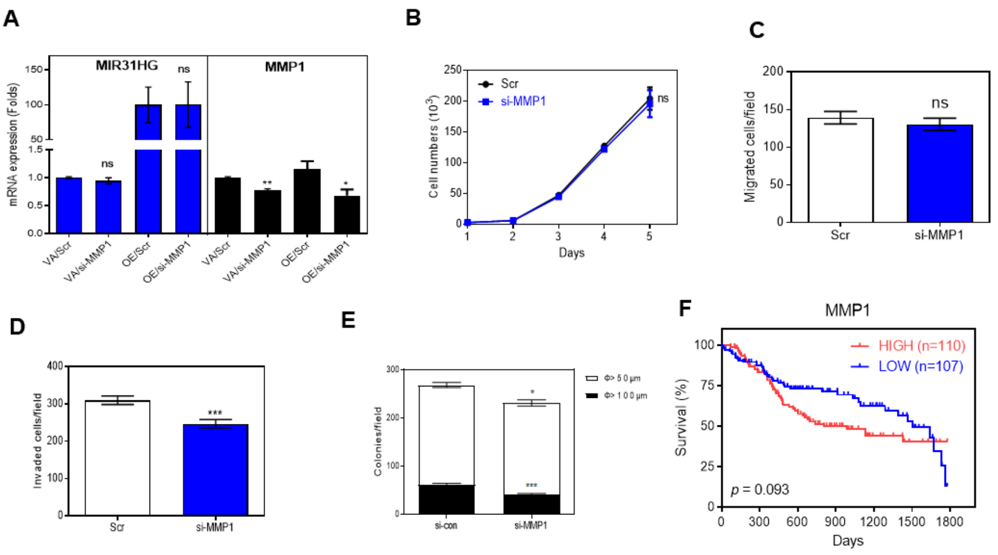

Figure S6. Knockdown of *MMP1* expression decreases invasion and colony formation by SAS cells. (A) qRT-PCR analysis. This indicates that there are slightly increases and decreases in *MMP1* mRNA expression following deletion of *MIR31HG* and knockdown of *MMP1*, respectively. (B – E) proliferation, migration, invasion and anchorage-independent colony formation assays. The knockdown of *MMP1* is associated with decreases in invasion and colony formation. (F) Survival analysis of HNSCC as related to *MMP1* expression in the TCGA tumor cohort. The tumors with *MMP1* expression in the highest quadrant exhibit a worse prognosis than their counterpart tumors in the lowest *MMP1* expression quadrant.

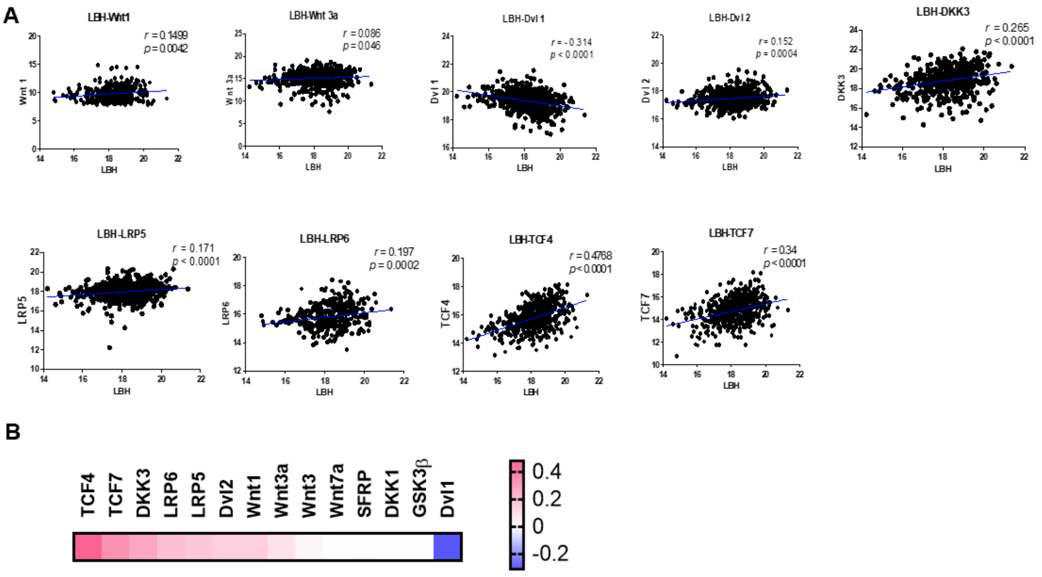

Figure S7. The correlation between *LBH* expression and the expression of genes involved in the Wnt signaling cascade in HNSCC tumors from the TCGA dataset. (A) Individual analysis of the expression of genes that are correlated with *LBH* expression. (B) Heatmap to illustrate the correlation. A correlation with *LBH* expression, mostly a positive correlation, was found for nine of the fourteen genes analyzed. *TCF4* and *TCF7* expression can be seen to be highly correlated with *LBH* expression. The expression of *Wnt3*, *Wnt7a*, *SFRP1*, *DKK1* and *GSK3β* is not significantly correlated with *LBH* expression. Gradient bar, correlation coefficient.

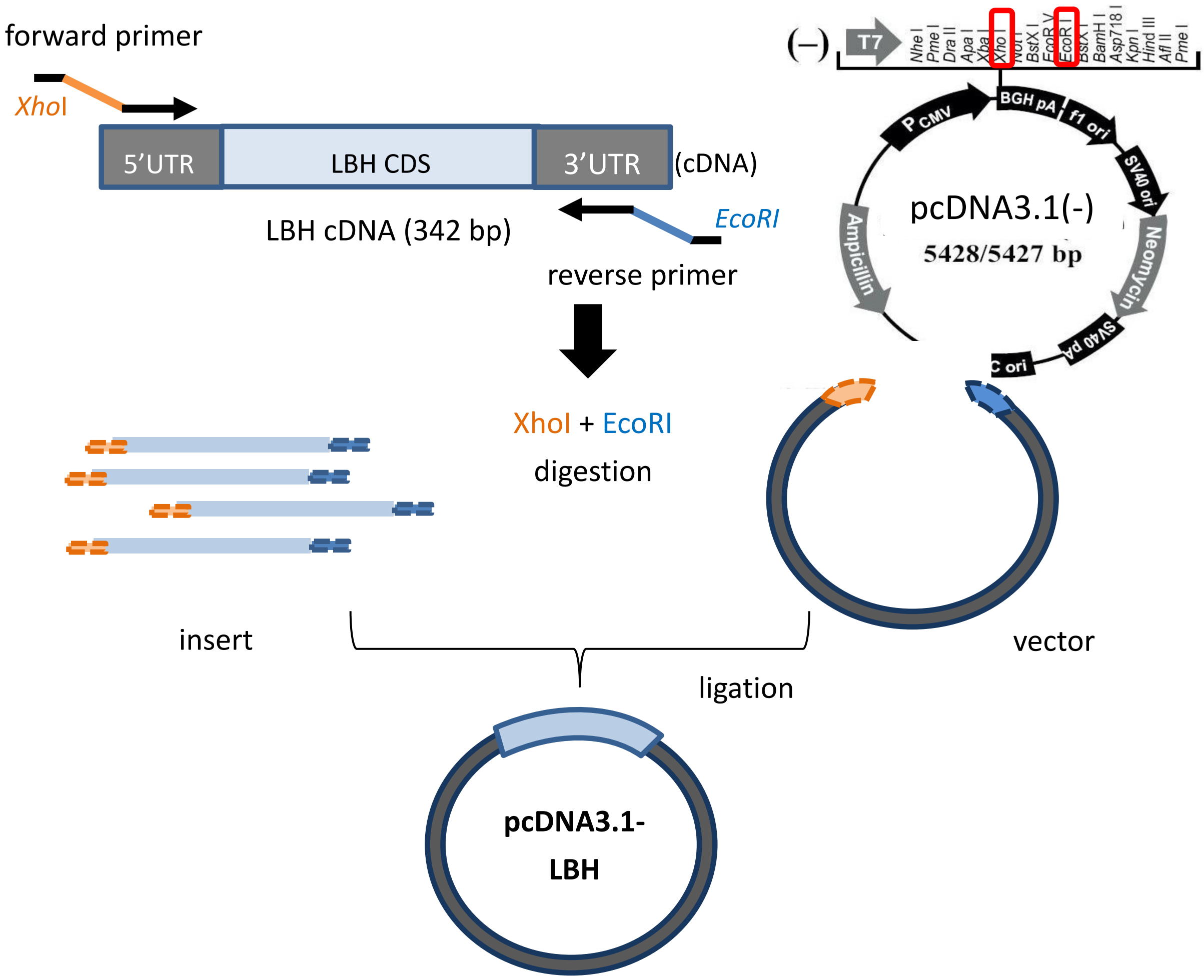

Figure S8. *LBH* transient overexpression plasmid. The strategy for the construction of the *LBH* plasmid for transient overexpression.

<u>**Supplementary Tables**</u>

Table S1. Clinicopathological characteristics of OPMD patients

		Patients for analysis (*n* = 25)*
Age (years)	51.4 ± 1.8	
Gender	Male	23
	Female	2
Histopathology	Epithelial hyperplasia and hyperkeratosis	12
	Epithelial dysplasia	16

*Three patients carry two individual lesions

Table S2. The primers used in this study to acquire PCR products for cloning

**Gene or construct**	**Direction**	**Sequences (5’ - 3’)**	**Amplicon (bps)**
MIR31HG	Forward 1	GATCCAGGTTCCACGTCCG	~2148
	Reverse 1	GTACCGACAAAGGTCACA AAGGATTTATTG	
MIR31HG	Forward 2	CAGGTTCCACGTCCGGCGC	~2148
	Reverse 2	CGACAAAGGTCACAAAGGATTTATTG	
LBH	Forward	GGGCTCGAGGCCCTAGGACTTCATGTC	342
	Reverse	CCCGAATTCCACAGGGACTCTACTGCTC	

Table S3. The candidate transcripts

Correlation	ID (ENSG 00000)	Gene name	Gene type	Gene description	FPKM			Ratio	
					Parental	KO	OE	KO	OE
Positive	265073	*AC010761.2*	antisense		0.12	0.05	0.35	0.4	2.9
	266947	*AC022916.1*	antisense		0.20	0.07	0.39	0.4	2.0
	226889	*AL359541.1*	antisense		0.32	0.14	0.64	0.4	2.0
	273129	*PACERR*	LncRNA	PTGS2 antisense NFKB1 complex-mediated expression regulator RNA	0.84	0.3	1.73	0.4	2.1
	243103	*RN7SL452P*	misc_RNA	RNA, 7SL, cytoplasmic 452, pseudogene	0.47	0.14	1.09	0.3	2.3
	231333	*RPL34P6*	processed_ pseudogene	ribosomal protein L34 pseudogene 6	0.27	0.12	0.77	0.4	2.9
	138135	*CH25H*	**protein_ coding**	cholesterol 25-hydroxylase	0.68	0.32	1.88	0.5	2.8
	125845	*BMP2*	**protein_ coding**	bone morphogenetic protein 2	0.47	0.2	1.07	0.4	2.3
	236925	*LTB*	**protein_ coding**	lymphotoxin beta	0.4	0.18	0.86	0.4	2.2
	258713	*C20orf141*	**protein_ coding**	chromosome 20 open reading frame 141	0.24	0.05	1.19	0.2	4.9
	175003	*SLC22A1*	**protein_ coding**	solute carrier family 22 member 1	0.2	0.09	0.41	0.4	2.1
	196611	*MMP1*	**protein_ coding**	matrix metallopeptidase 1	99.31	34.73	282.01	0.3	2.8
	166532	*RIMKLB*	**protein_ coding**	ribosomal modification protein rimK like family member B	0.20	0.08	0.81	0.4	4.1
	151229	*SLC2A13*	**protein_ coding**	solute carrier family 2 member 13	0.21	0.10	0.56	0.5	2.7
	105427	*CNFN*	**protein_ coding**	cornifelin	0.38	0.14	0.79	0.4	2.1
	189221	*MAOA*	**protein_ coding**	monoamine oxidase A	0.44	0.10	1.03	0.2	2.3
	138646	*HERC5*	**protein_ coding**	HECT and RLD domain containing E3 ubiquitin protein ligase 5	0.36	0.17	3.04	0.5	8.5
	107551	*RASSF4*	**protein_ coding**	Ras association domain family member 4	0.12	0.06	0.24	0.5	2.1
	221303	*SNORA79*	snoRNA	small nucleolar RNA, H/ACA box 79	0.66	0.29	2.64	0.4	4.0

	199805	*RNU1-134P*	snRNA	RNA, U1 small nuclear 134, pseudogene	0.56	0.25	1.28	0.4	2.3
	229344	*MTCO2P12*	unprocessed_ pseudogene	mitochondrially encoded cytochrome c oxidase II pseudogene 12	0.13	0.06	0.31	0.4	2.3
	266191	*RF00017*			2.46	0.41	6.18	0.2	2.5
**Negative**	269019	*HOMER3-AS1*	antisense	HOMER3 antisense RNA 1	0.19	0.43	0.06	2.2	0.3
	263627	*PPP4R1-AS1*	LncRNA	PPP4R1 antisense RNA 1	0.36	0.76	0.14	2.1	0.4
	272993	*AC239868.3*	LncRNA		0.68	1.4	0.24	2	0.3
	242175	*RN7SL127P*	misc_RNA	RNA, 7SL, cytoplasmic 127, pseudogene	0.46	1.36	0.18	3.0	0.4
	199459	*RF00019*	misc_RNA		1.25	3.35	0.48	2.7	0.4
	233558	*AL050331.1*	processed_ pseudogene		0.66	1.51	0.25	2.3	0.4
	263829	*SINHCAFP1*	processed_ pseudogene	SINHCAF pseudogene 1	0.21	0.68	0.08	3.3	0.4
	049769	*PPP1R3F*	**protein_ coding**	protein phosphatase 1 regulatory subunit 3F	0.84	1.69	0.34	2.0	0.4
	138061	*CYP1B1*	**protein_ coding**	cytochrome P450 family 1 subfamily B member 1	0.23	0.49	0.1	2.1	0.4
	116039	*ATP6V1B1*	**protein_ coding**	ATPase H+ transporting V1 subunit B1	0.17	0.43	0.08	2.5	0.5
	213626	*LBH*	**protein_ coding**	limb bud and heart development	5.04	12.74	2.08	2.5	0.4
	029534	*ANK1*	**protein_ coding**	ankyrin 1	0.42	0.9	0.18	2.2	0.4
	214773	*AC112512.1*	sense_intronic		0.16	0.34	0.07	2.1	0.5
	207187	*SNORA10B*	snoRNA	small nucleolar RNA, H/ACA box 10B	1.04	2.77	0.4	2.7	0.4
	279691	*AC113410.3*	TEC		1.58	3.83	0.52	2.4	0.3
	240785	*RPL36AP21*	transcribed_ processed_ pseudogene	ribosomal protein L36a pseudogene 21	0.21	0.45	0.08	2.1	0.4
	248352	*TNXA*	pseudogene	tenascin XA (pseudogene)	0.23	0.48	0.05	2.1	0.2
	282079	*TAS2R64P*	pseudogene	taste 2 receptor member 64 pseudogene	0.15	0.44	0.06	3.0	0.4
	283989	*AC270107.2*	not defined		1.49	4.65	0.29	3.1	0.2

Table S4. The candidate genes

Effector	Gene name	FPKM			Ratio	
		Parental	KO	OE	KO	OE
Positive	*HERC5*	0.36	0.17	3.04	0.5	8.5
	C20orf141	0.24	0.05	1.19	0.2	4.9
	RIMKLB	0.20	0.08	0.81	0.4	4.1
	MMP1	99.31	34.73	282.01	0.3	2.8
	CH25H	0.68	0.32	1.88	0.5	2.8
	SLC2A13	0.21	0.10	0.56	0.5	2.7
	BMP2	0.47	0.20	1.07	0.4	2.3
	MAOA	0.44	0.10	1.03	0.2	2.3
	LTB	0.40	0.18	0.86	0.4	2.2
	SLC22A1	0.20	0.09	0.41	0.4	2.1
	CNFN	0.38	0.14	0.79	0.4	2.1
	RASSF4	0.12	0.06	0.24	0.5	2.1
Negative	*ATP6V1B1*	0.17	0.43	0.08	2.5	0.5
	LBH	5.04	12.74	2.08	2.5	0.4
	ANK1	0.42	0.90	0.18	2.2	0.4
	CYP1B1	0.23	0.49	0.10	2.1	0.4
	PPP1R3F	0.84	1.69	0.34	2.0	0.4

Table S5. siRNAs and control oligonucleotide used in this study

siRNA	Supplier	Cat. No.
Scr	Ambion	4390843
	BioTools	siRNA Negative Control
si-*MIR31HG*	Ambion	n266049
si-*MMP1*	BioTools	MMP1-homo-1071
		MMP1-homo-338
si-*LBH*	Ambion	s37687

Table S6. The TaqMan probes used in this study

probe	Supplier	Cat. No.
MIR31HG	Applied biosystems	Hs01007339_g1
MMP1	Applied biosystems	Hs00899658_m1
SLC2A13	Applied biosystems	Hs01573078_m1
BMP2	Applied biosystems	Hs00154192_m1
LBH	Applied biosystems	Hs00368853_m1
GAPDH	Applied biosystems	Hs00266705_g1
miR-31	Applied biosystems	002279
RNU6B	Applied biosystems	001093

Table S7. The primary antibodies used in this study

Antibody	MW (kDa)	Host	Dilution	Supplier	Cat. No.
**GAPDH**	36	mouse	1: 10000	Santa Cruz Biotech	sc32233
**BMP2**	60	goat	1:500	Santa Cruz Biotech	sc6895
**MMP1**	54	rabbit	1:5000	Abcam	ab38929
**LBH**	17	rabbit	1:1000	Invitrogen	PA572031
**FAK**	125	rabbit	1:1000	Santa Cruz Biotech	sc557
**p-FAK (tyr-925)**	125	goat	1:1000	Santa Cruz Biotech	sc11766
**AKT1**	60	mouse	1:1000	Santa Cruz Biotech	sc5298
**p-AKT (thr-308)**	60	rabbit	1:1000	Cell Signaling	4056
**p44/42 MAPK (ERK1/2)**	42,44	rabbit	1:1000	Cell Signaling	9102
**p-p44/42 MAPK (ERK1/2) (Thr202/Tyr204)**	42,44	rabbit	1:1000	Santa Cruz Biotech	9101
**src**	60	mouse	1:1000	Cell Signaling	2110
**p-src (Y418)**	60	rabbit	1:1000	Biosource	411223A
**Active β-catenin**	92	mouse	1:1000	Millipore	05665
**p-GSK3α/β (ser-21/9)**	46	rabbit	1:1000	Cell Signaling	9311L
**TCF4**	68	rabbit	1:1000	Epitomics	21141

Table S8. The secondary antibodies used in this study.

Antibody	Dilution	Supplier	Cat. No.
**anti-goat**	1:1000	Millipore	AP106P
**anti-mouse**	1:1000	Millipore	AP124P
**anti-rabbit**	1:1000	Millipore	AP132P
